# Neuroimmune Modulation Through Vagus Nerve Stimulation Reduces Inflammatory Activity in Crohn’s Disease Patients: A Prospective Open-label Study

**DOI:** 10.1093/ecco-jcc/jjad151

**Published:** 2023-09-21

**Authors:** Geert D’Haens, Michael Eberhardson, Zeljko Cabrijan, Silvio Danese, Remco van den Berg, Mark Löwenberg, Gionata Fiorino, P Richard Schuurman, Göran Lind, Per Almqvist, Peder S Olofsson, Kevin J Tracey, Stephen B Hanauer, Ralph Zitnik, David Chernoff, Yaakov A Levine

**Affiliations:** Department of Gastroenterology and Hepatology, Amsterdam UMC, Amsterdam, The Netherlands; Department of Medicine, Karolinska Institutet, Solna, Sweden; Department of Health, Medicine and Caring Sciences, Linköping University, Linköping, Sweden; Division of Gastroenterology, Hepatology and Clinical Nutrition, University Hospital Dubrava, Zagreb, Croatia; Division of Gastroenterology, University of Applied Health Sciences, Zagreb, Croatia; Josip Juraj Strossmayer University of Osijek School of Medicine, Osijek, Croatia; Department of Gastroenterology and Endoscopy, IRCCS Ospedale San Raffaele, Italy; Department of Gastroenterology and Endoscopy, University Vita-Salute San Raffaele, Milano, Italy; Department of Gastroenterology and Hepatology, Amsterdam UMC, Amsterdam, The Netherlands; Department of Gastroenterology and Hepatology, Amsterdam UMC, Amsterdam, The Netherlands; Department of Gastroenterology and Digestive Endoscopy, VIta-Salute San Raffaele Hospital, Milan, Italy; IBD Unit, Department of Gastroenterology and Digestive Endoscopy, San Camillo-Forlanini Hospital, Rome, Italy; Department of Neurosurgery, Amsterdam UMC, Amsterdam, The Netherlands; Department of Clinical Neuroscience, Karolinska Institutet, Stockholm, Sweden; Department of Neurosurgery, Karolinska University Hospital, Stockholm, Sweden; Department of Clinical Neuroscience, Karolinska Institutet, Stockholm, Sweden; Department of Neurosurgery, Karolinska University Hospital, Stockholm, Sweden; Neurosurgery Stockholm AB, Stockholm, Sweden; Department of Medicine, Solna, Karolinska Institutet, Karolinska University Hospital, Stockholm, Sweden; Feinstein Institutes for Medical Research, Manhasset, New York; Feinstein Institutes for Medical Research, Manhasset, New York; Department of Neurosurgery, Donald and Barbara Zucker School of Medicine at Hofstra/Northwell, Hempstead, New York, USA; Department of Molecular Medicine, Donald and Barbara Zucker School of Medicine at Hofstra/Northwell, Hempstead, New York, USA; Division of Gastroenterology and Hepatology, Northwestern University–Feinberg School of Medicine, Chicago, Illinois, USA; SetPoint Medical, Valencia, California, USA; Valerio Consulting, Santa Barbara, California, USA; SetPoint Medical, Valencia, California, USA; Department of Medicine, Karolinska Institutet, Solna, Sweden; Department of Molecular Medicine, Donald and Barbara Zucker School of Medicine at Hofstra/Northwell, Hempstead, New York, USA; SetPoint Medical, Valencia, California, USA

**Keywords:** Crohn’s disease, vagus nerve, neuroimmune modulation

## Abstract

**Background and Aims:**

Crohn’s disease [CD] is a debilitating, inflammatory condition affecting the gastrointestinal tract. There is no cure and sustained clinical and endoscopic remission is achieved by fewer than half of patients with current therapies. The immunoregulatory function of the vagus nerve, the ‘inflammatory reflex’, has been established in patients with rheumatoid arthritis and biologic-naive CD. The aim of this study was to explore the safety and efficacy of vagus nerve stimulation in patients with treatment-refractory CD, in a 16-week, open-label, multicentre, clinical trial.

**Methods:**

A vagus nerve stimulator was implanted in 17 biologic drug-refractory patients with moderately to severely active CD. One patient exited the study pre-treatment, and 16 patients were treated with vagus nerve stimulation [4/16 receiving concomitant biologics] during 16 weeks of induction and 24 months of maintenance treatment. Endpoints included clinical improvement, patient-reported outcomes, objective measures of inflammation [endoscopic/molecular], and safety.

**Results:**

There was a statistically significant and clinically meaningful decrease in CD Activity Index at Week 16 [mean ± SD: -86.2 ± 92.8, *p* = 0.003], a significant decrease in faecal calprotectin [-2923 ± 4104, *p* = 0.015], a decrease in mucosal inflammation in 11/15 patients with paired endoscopies [-2.1 ± 1.7, *p* = 0.23], and a decrease in serum tumour necrosis factor and interferon-γ [46–52%]. Two quality-of-life indices improved in 7/11 patients treated without biologics. There was one study-related severe adverse event: a postoperative infection requiring device explantation.

**Conclusions:**

Neuroimmune modulation via vagus nerve stimulation was generally safe and well tolerated, with a clinically meaningful reduction in clinical disease activity associated with endoscopic improvement, reduced levels of faecal calprotectin and serum cytokines, and improved quality of life.

## Graphical Abstract

**Graphical Abstract:**
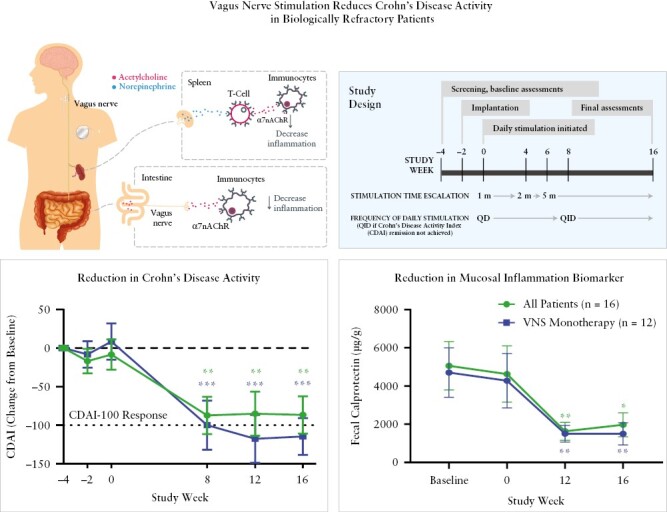

## 1. Introduction

Crohn’s disease [CD] is a chronic inflammatory condition that can affect any portion of the gastrointestinal tract. The incidences of CD in the USA and in Europe are approximately 10.7 and 6.3 per 100 000 person-years, respectively, with a trending increase in incidence as well as prevalence.^1–3^ Current treatments include corticosteroids, immunomodulators, and biologic drugs including anti-tumour necrosis factor [TNF] antibodies such as infliximab and adalimumab. Other biologics include anti-interleukin [IL]-12/IL-23 antibodies and the integrin-inhibiting antibody vedolizumab, as second-line therapeutics. However, many patients do not have an adequate response or develop a loss of response. On average, 65% of patients with severe CD fail to attain steroid-free clinical remission.^4–7^ Furthermore, biologics and conventional therapies for moderately to severely active CD have potential significant side effects, and some include black box warnings for infections or malignancies.

**Figure 1.**
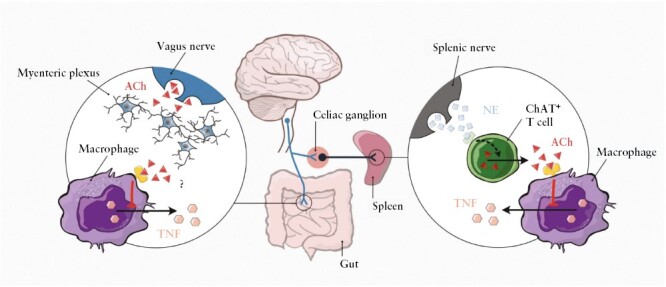
The inflammatory reflex. The vagus nerve functionally projects into the coeliac plexus which relays signals to the sympathetic splanchnic nerve. Activation promotes release of acetylcholine [ACh] from choline acetyltransferase [ChAT]-expressing T cells which inhibits the release of tumour necrosis factor [TNF] from splenic macrophages. Vagus nerve endings also project into the gut wall and interface with the enteric nervous system through the myenteric plexus, but the role of ChAT^+^ T cells in the gut has not yet been fully elucidated. NE, norepinephrine/noradrenaline. Reprinted from *International Immunology* 2021;**33**:349–56].

The vagus nerve, a bilateral cranial nerve which arises in the brain stem and innervates the body’s organs, is an established therapeutic target for patients with drug-refractory epilepsy and depression.^8^ It communicates bidirectional information between the viscera and central nervous system through afferent and efferent neurons [approximately 80% and 20% of neurons, respectively]^9^ [Figure 1]. For the past three decades, electrical devices to stimulate the vagus nerve have been implanted in more than 125 000 patients to treat drug-refractory epilepsy and depression.^8^ This procedure is established to be safe, well tolerated, and devoid of significant long-term complications.^10^

Vagus nerve fibres also mediate the ‘inflammatory reflex’, an innate neuroimmune mechanism that is responsive to and inhibits inflammation in the intestines and other organs.^11^ Cytokine release in tissues stimulates the sensory arm of the inflammatory reflex, which relays cytokine-specific action potentials to the brainstem.^12,13^ Arrival of these signals activates brain stem motor neurons that reflexively transmit signals via the vagus nerve back to the organs. These vagus motor signals inhibit cytokine release in tissues via the myenteric plexus to the gut and the coeliac plexus to the spleen.^14–19^ The downstream mechanism of the inflammatory reflex is mediated by signal transduction via the α7 nicotinic acetylcholine [ACh] receptors on immunocytes which inhibit nuclear factor kappa B [NF-κB], Janus kinase [JAK]/signal transducer and activator of transcription proteins [STAT], and inflammasome activation.^20–22^ Other anti-inflammatory mechanisms of vagus nerve stimulation include suppressing CD11b expression, increasing T regulatory cells, decreasing Th1 cells, reducing auto-antibody production, and increasing specialised, proresolving mediator release^23–29]^.

Recent preclinical and clinical studies of electrical stimulation of the vagus nerve indicate significant attenuation of experimental colitis and CD signs and symptoms.^18,30–36^ A pilot, single-centre, clinical study of seven biologic-naïve CD patients used an implanted vagus nerve stimulator programmed with parameters used to treat epilepsy. CD activity was reduced in five [71%] patients and four patients achieved clinical and endoscopic remission within 6 months.^36^ In a 1-year follow up, and including two additional subjects, both clinical and endoscopic remission were observed in five patients at 12 months.^37^ The stimulation parameters [500 µs pulse width and 10 Hz] were adjusted during the trial to reach maximum tolerated intensities ranging between 0.25 mA and 1.25 mA, in cycles which ran 24 h per day for 30 s on followed by 5 min off. Recently, we observed that delivering much lower charge [250 µs pulse width, 10 Hz] for only 1–4 minutes per day was sufficient to significantly reduce inflammation and disease severity in preclinical animal models and in patients with rheumatoid arthritis.^34,38,39^

Despite this evidence, it was previously unknown whether stimulation of the vagus nerve with lesser electrical charge and reduced daily frequency [only once to four times daily] would decrease CD severity in biologic-refractory patients. Herein, we provide the results from a 16-week, open-label, multicentre, clinical trial with clinical and objective endpoints investigating the safety and efficacy of vagus nerve stimulation by an implanted electrical pulse generator in patients with moderately to severely active CD and insufficient or absent response to biologic drugs.

## 2. Materials and Methods

### 2.1. Study design and participants

We performed a 16-week, multicentre, open-label trial in four EU countries, examining the safety and efficacy of electrical stimulation of the vagus nerve as an innovative treatment for CD. Patients were 18–75 years of age, with moderately to severely active refractory CD >4 months after diagnosis and with a Crohn’s Disease Activity Index [CDAI] 220 to 450, endoscopic evidence of ulceration by Simple Endoscopic Score for Crohn Disease [SES-CD ulcer size score of at least 2 in at least one segment], and faecal calprotectin concentrations greater or equal to 200 μg/g.

Patients were eligible for enrolment if they had an insufficient response or were intolerant to at least one TNF inhibitor [ie, infliximab or adalimumab] or vedolizumab. Patients in Sweden were required to have failed both an anti-TNF agent and vedolizumab prior to enrolment. Azathioprine, 6-mercaptopurine, and methotrexate could be continued throughout the study but had to be stable for >12 weeks prior to enrolment. Prohibited medications within the pre-enrolment washout window included any TNF inhibitor, natalizumab, vedolizumab, oral glucocorticoids at doses greater than 10 mg prednisone orally daily, or an equivalent dose of other oral or parenteral glucocorticoids within 4 weeks, ciclosporin, tacrolimus, sirolimus, or mycophenolate mofetil within 4 weeks, intravenous antibiotics for CD within 4 weeks, parenteral or enteral feeding, or elemental diet within 2 weeks, and rectal use of 5-aminosalicylates or corticosteroid enemas or suppositories within 2 weeks. During the study [after the first nine patients were enrolled], a protocol modification was made that allowed for continued use of a stable [for at least 6 months] dose of a TNF inhibitor or vedolizumab throughout the study, to limit the amount of time the patients were untreated prior to Day 0. Exclusion criteria included coeliac disease, ulcerative or indeterminate colitis, enterocutaneous fistulae with abscesses, extensive colonic resection, bowel-related surgery within 12 weeks prior to enrolment, prior vagotomy, history of vasovagal syncope, pharyngeal dysfunction, preexisting vocal cord damage or dysfunction, uncontrolled asthma or obstructive lung disease, peptic ulcer disease, significant cardiac rhythm disturbances, sleep apnoea, or the use of other electrically active medical devices. A full list of inclusion and exclusion criteria can be found in Supplementary Table 1.

The safety-evaluable population included all patients who were screened, and in this group all adverse events were reported beginning at the time of signed informed consent. The efficacy-evaluable population included all patients who were implanted with the vagus nerve stimulation device and for whom at least one post-implantation documentation of primary efficacy data was available.

### 2.2. Intervention

**Figure 2.**
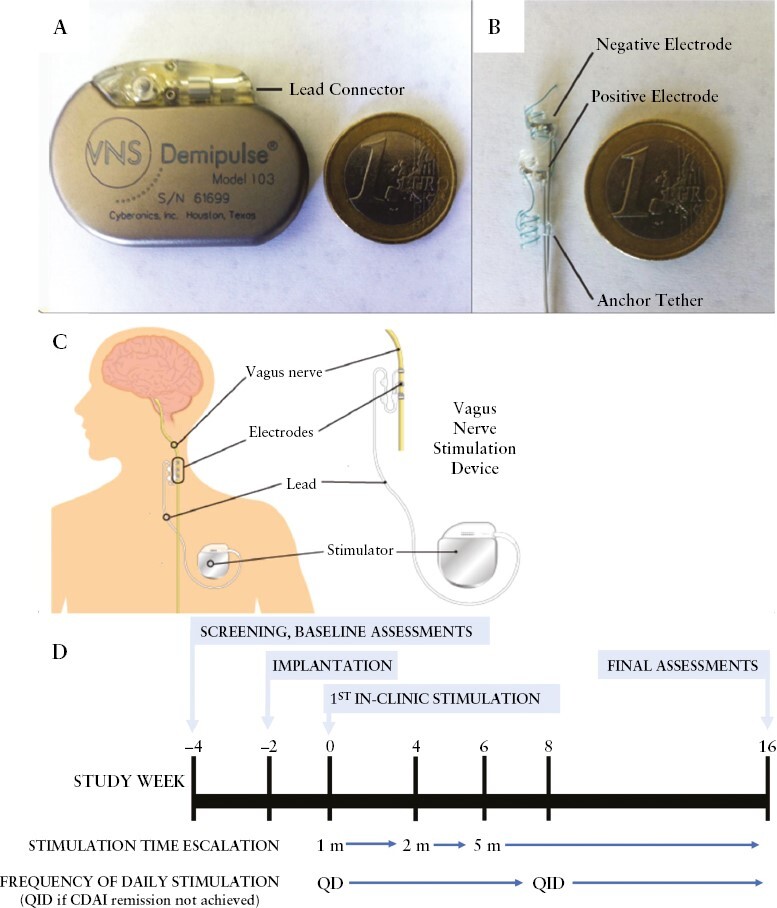
Study device and design.

The investigational study device was a standard Cyberonics VNS Therapy® System, including an implanted pulse generator (Demipulse Model 103; Cyberonics [now Livanova], London, UK) that was placed in a subcutaneous pocket in the chest wall, and lead [PerenniaFLEX Lead Model 304] that was secured around the left cervical vagus nerve and tunnelled to the pulse generator [Figure 2A–C].^40^ The external programming system includes the programming wand, the programming software, a compatible computer, and an actuating magnet. This software allows a physician to place the programming wand over the pulse generator to read and change device parameters. The automatic stimulation [optimised for epilepsy] was disabled on the device and the magnet was provided to the patients, with instructions for use for actuating the device daily according to protocol. The system components were treated as investigational study devices due to their off-label use in patients with CD.

The device settings chosen for this study were based on extensive preclinical studies, the proof-of-concept clinical study that was performed with the same device in two rheumatoid arthritis cohorts, as well as clinical vagus nerve stimulation experience with these devices used in epilepsy and depression.^39,41,42^ The study used a pulse frequency and pulse duration of 10 Hz and 250 µs, respectively, well below the specified upper limits allowed in the currently approved vagus nerve stimulation device product labelling in approved indications of drug-refractory epilepsy and depression.^43^

The study flow chart is shown in Figure 2D. Patients had screening assessments and baseline clinical and biomarker assessments at the Week -4 visit and the Week 2 visit, after which the device was implanted under general anaesthesia at the Week 2 Visit. During the implantation procedure prior to wound closure, the patients received a single stimulus as part of the standard intraoperative diagnostic testing to check system function and lead integrity and impedance. The device was then inactivated and the patient allowed recovery from surgery for at least 14 days. On the Week 0 visit, patients had postoperative clinical assessments and the stimulation was titrated to an output current as maximally tolerated between a minimum of 0.25 mA and a maximum of 2.0 mA [in 0.25-mA increments]. The patients self-administered for 1 min once per day active stimulation at a pulse frequency of 10Hz, a 250-µs pulse duration daily by passing the actuating magnet across their chest. On each visit between Weeks 1 and 4, the stimulation output current was further incremented as tolerated. At the Week 4 visit, daily stimulation time was increased to 2 min and at the Week 6 visit to 5 min. At Week 8 clinical scores were assessed, and if the patient had not achieved remission [CDAI <150], daily stimulation time was increased to 5 min four times per day. Early termination visits were analysed as Week 16 visits.

At the conclusion of the study, patients were offered the option to have the device surgically removed, left in place and inactivated, or to continue treatment in a long-term extension study that ends when the last patient enrolled reaches the 24-month study visit. Eleven of 16 patients in the efficacy group opted to continue in the extension study, which will be reported separately.

### 2.3. Assessments and measurements

#### 2.3.1. Efficacy measures

The primary efficacy endpoint was mean change in CDAI between the preimplantation baseline and the Week 16 visit. Other endpoints included the proportion of patients that achieved CDAI remission [CDAI <150] and response [CDAI -70; drop in CDAI by at least 70 points] or enhanced response [CDAI -100; drop in CDAI by at least 100 points]. CDAI with partially missing CDAI subscores were calculated with the missing subscore imputed by last value carried forward. No CDAI imputations were allowed for study visits missing all CDAI subscore values or carried forward from a pre- to a post-therapy initiated time point.

Endoscopy was performed at screening and at the Week 16 visit and biopsies were collected. Recorded endoscopic videos were scored by two expert central readers blinded to the timing of the recordings. The averaged value of the independent scores was used for the primary analysis of SES-CD. SES-CD remission was defined as all observed segments having an ulcer score of </= 1. Standard forceps biopsies were collected from involved regions of ileum and colon and formalin fixed for standard histology. The most affected regions were read at a central reading facility by a pathologist blinded to the patient and treatment sequence information, using the semi-quantitative Geboes Score.^44,45^

Biomarker endpoints included change from baseline in faecal calprotectin, high-sensitivity C-reactive protein [hsCRP], and serum cytokines, and the heart rate variability-derived autonomic balance. Faecal calprotectin and hsCRP were measured centrally and serum cytokines were measured by MSD electrochemiluminescence [MSD Chemokine Panel 1 and Proinflammatory Panel 1, Meso Scale Discovery, Rockville, MD, USA].

Two quality-of-life scales were used to assess health-related quality of life: the Inflammatory Bowel Disease Questionnaire [IBDQ; MID of 16] and the Simple Health Score [SHS] instruments.^46^ The IBDQ scale increases and the SHS decreases as patients improve.

#### 2.3.2. Safety measures

The overall safety and tolerability of the implantation, device, and treatment were assessed for the safety-evaluable population. Safety endpoints included were incidence, causality, and severity of serious adverse events [SAEs], adverse events, and clinical laboratory results. These were assessed throughout the study, coded using the Medical Dictionary for Regulatory Activities [MedDRA], and are presented by MedDRA term as incidence rates.

#### 2.3.3. Statistical analyses

Descriptive statistics and the 95% confidence intervals [CIs] of the mean difference from baseline were calculated for CDAI, SES-CD, faecal calprotectin, hsCRP, IBDQ, and SHS. Changes from baseline to the Week 16 primary endpoint were compared by paired t test. As this was a pilot study, no adjustment for multiple comparisons were prespecified. In post hoc analyses, changes in CDAI, faecal calprotectin, and hsCRP from baseline to each study visit were further tested with a paired, mixed-effects analysis of variance [ANOVA] model [Restricted Maximum Likeliness; REML], adjusted with Bonferroni’s multiple comparisons test [Prism V.9, GraphPad, San Diego, CA, USA], and included in the figures. Changes in serum cytokine levels were assessed by Wilcoxon matched pairs signed rank test. Endoscopy across the SES-CD subscores and histopathology across the Geboes subscores were calculated by REML adjusted with Bonferroni’s multiple comparisons test. Correlations between continuous or discrete clinical, molecular, endoscopic, and quality-of-life parameters were quantified using Spearman’s rank correlation coefficients [Prism V.9, GraphPad]. In further post hoc analyses of the patients achieving a clinical response to therapy, median changes in clinical and patient-reported outcomes [CDAI, IBDQ, SHS] and in objective outcomes [faecal calprotectin, hsCRP, SES-CD, and the SES-CD colon subcomponent] were assessed by Wilcoxon column test vs a hypothetical change of 0.

### 2.4. Ethics statement

This study was done in accordance with the International Conference on Harmonisation guidelines for good clinical practice and the ethical principles of the Declaration of Helsinki. All patients gave written informed consent, which was reviewed and approved by an independent ethics committee or institutional review board. The study was registered with clinicalTrials.gov [NCT02311660].

## 3. Results

### 3.1. Patient disposition and baseline characteristics

**Figure 3.**
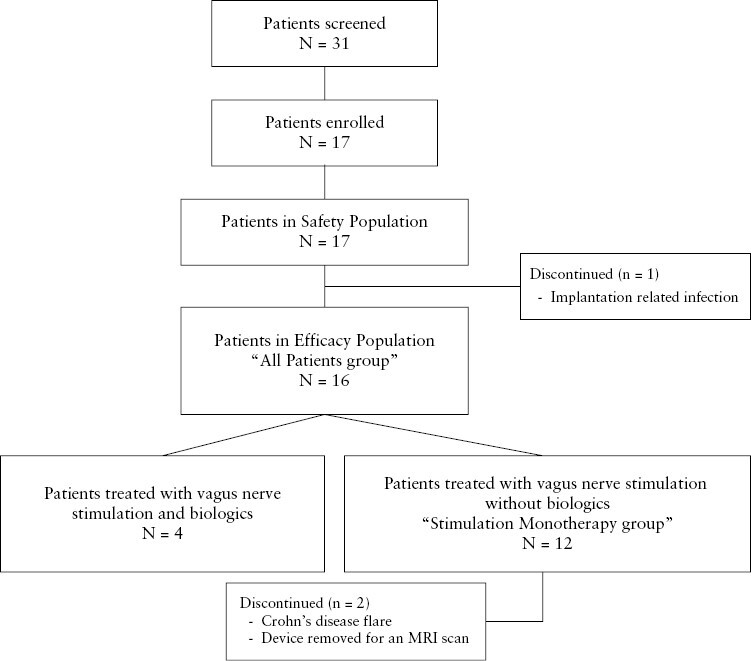
Patient disposition.

A total of 31 patients were screened and 17 patients with active CD were enrolled at five sites and received a vagus nerve stimulator system implanted on the left cervical vagus nerve [Figure 2A–C]. Sixteen [94%] were included in the efficacy population because one patient suffered a postoperative wound infection, and the device was removed before stimulation had commenced [‘All Patients group’]. Twelve of the 16 patients [designated as ‘Stimulation Monotherapy group’] were analysed separately because the only nonconventional treatment they received during the study period was vagus nerve stimulation. Four patients continued biologic treatment during the 16-week study, at the treating physician’s discretion [2/4 treated on a stable dose]. Eleven of 17 subjects had any exposure to a corticosteroid during the trial, and 5 of 17 received >10 mg at any point after treatment was initiated on Day 0. Two patients in the efficacy population withdrew from the study prior to Week 16, one due to a CD flare and one to undergo a magnetic resonance imaging [MRI] scan that was incompatible with the specific device implanted [Figure 3].

The mean [range] age of the 17 patients at baseline was 35.4 [21–62] years, mean [range] body mass index [BMI] of patients was 22 [16.8–29.0], 76.5% of patients were male, and 82.4% of patients were White. The mean [range] number of biologic drugs previously experienced was 2.2 [1–6]. Patient characteristics are summarised in Table 1.

**Table 1.** Baseline characteristics.

Total Safety Population, *n*	17
Enrolment by country	
The Netherlands	6
Croatia	7 [6 in Efficacy Population]
Sweden	2
Italy	2
Sex, *n* [% male]	13 [76.5]
Ethnicity, *n* [% Caucasian]	14 [82.4]
Mean age, years [range]	35.4 [21.0–62.0]
Mean age at CD diagnosis, years [range]	23.5 [14.2–57.0]
Mean CD duration, years [range]	12.2 [4.7–25.2]
Mean height, cm [SD; range]	174.7 [5.8; 162.5–186.0]
Mean weight, kg [SD; range]	67.1 [11.7; 50.0–88.7]
Mean BMI, kg/m^2^ [SD; range]	22.0 [4.0; 16.8–29.0]
Number of prior biologics [SD; range]	2.2 [1.4; 1–6]
Number of patients on biologics at baseline	4
Number of patients with prior bowel resection	7
Number of patients with prior or current perianal fistula	5
Number of patients with confirmed colonic involvement	17
Number of patients with confirmed ileal involvement	11
Total Efficacy Population, *n*	16
CDAI [SD]	306.4 [59.4]
SES-CD [SD]	20.2 [6.8]
Faecal calprotectin [SD]	5054.4 [5062.54]
High-sensitivity CRP, mg/L [SD]	4.4 [3.4]

Abbreviations: BMI: Body Mass Index, CD = Crohn’s Disease, CDAI = Crohn’s Disease Activity Index, CRP = C-Reactive Protein.

### 3.2. Efficacy endpoints

**Figure 4.**
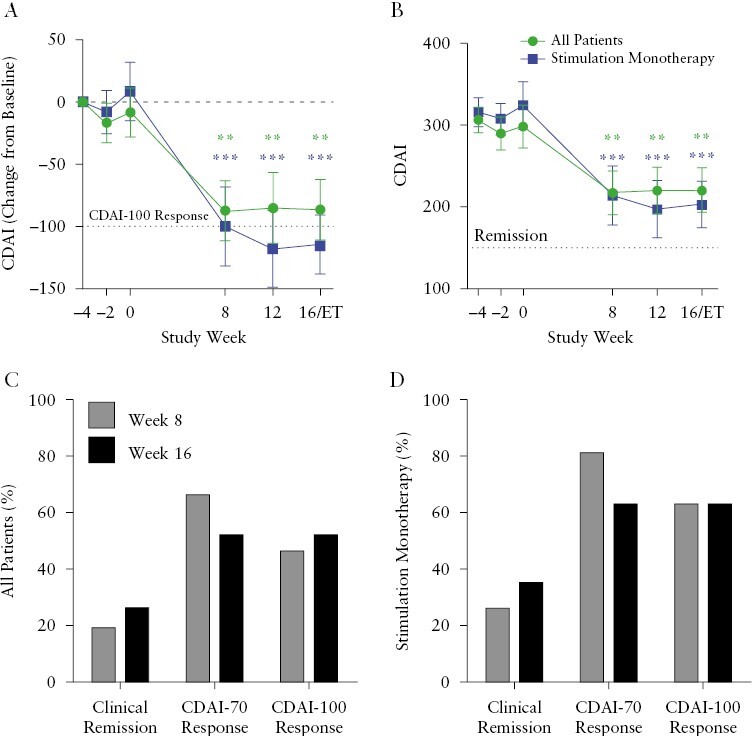
Clinical efficacy. [A] Change from baseline in CDAI [mean ± SEM] and [B] CDAI [mean ± SEM] over time in the All Patients Efficacy population [*n* = 16] and in the 12-patient Stimulation Monotherapy subpopulation. [C] Percent of All Patients or [D] Stimulation Monotherapy patients who achieved clinical remission [CDAI <150], CDAI-70 [CDAI decrease from baseline ≥70], and CDAI-100 [CDAI decrease from baseline ≥100]. CDAI and its change from baseline were analysed with a paired mixed-effects model [restricted maximum likeliness; REML] and adjusted with Bonferroni’s multiple comparisons test. **p <0.01, ***p <0.001. CDAI, Crohn’s Disease Activity Index; SEM, standard error of the mean.

Mean clinical disease activity was stable from the screening visit [Week -4] to Week 0 until the onset of daily stimulation of the vagus nerve. After the onset of vagus nerve stimulation, the mean decrease in CDAI from baseline to Week 16 was (mean ± standard error of the mean [SEM]: -86.2 ± 24.3, *p* = 0.003] in the full cohort, and -114.5 ± 23.9, [*p* = 0.0002] in the Stimulation Monotherapy group [Table 2, Figure 4A]. The individual patient change in CDAI during the study is shown in Supplementary Figure 1A and B. During active treatment, mean CDAI decreased from baseline ([mean ± SEM] in the All Patients group: baseline: 306 ± 15; Week 8: 218 ± 29, *p *= 0.001; Week 16: 221 ± 27, *p *= 0.003) [Figure 4B]. Clinical remission [CDAI <150] was achieved in 27% of the All Patients group and 36% of the Stimulation Monotherapy group, respectively, at Week 16. By Week 8, 47% and 64% of patients met the CDAI-100 criterion and more than half of those treated [53% and 64% of the All Patients group and Stimulation Monotherapy group, respectively] met the CDAI-100 threshold at Week 16. The individual patient CDAI scores are plotted in Supplementary Figure 2A and B. The proportions of patients who improved sufficiently to achieve the definition of clinical remission, and the CDAI-70 and CDAI-100 clinical responses following 8 and 16 weeks of vagus nerve stimulation treatment, are shown in Figure 4C and D.

**Table 2.** Efficacy statistics.

Week 16 change from Baseline	*N*	Mean	Std	Min	Q1	Median	Q3	Max	Lower 95% CI	Upper 95% CI	T-test *p*-value
CDAI	15^1^	-86.2	92.79	-249	-160	-121	-7	101	-137.6	-34.8	0.003
SES-CD	15	-2.1	6.43	-11	-6.5	-3	0.5	15	-5.6	1.5	0.23
IBDQ	15	9.4	34.14	-41	-23	7	46	60	-9.5	28.3	0.3
SHS	15	-33.7	107.74	-240	-121	-39	68	128	-93.3	26	0.25
Faecal calprotectin [µg/g]	15	-2923	4104	-10871	-5357	-1623	-4622	4395	-5196	-650	0.015
hsCRP [mg/dL]	16	-0.5	2.71	-4.7	-2.3	-0.4	0.4	5.5	-2	0.9	0.46

^1^Week 16 or Early Termination samples not available for every patient.

Abbreviations: CDAI = Crohn’s Disease Activity Index, hsCRP = High-Sensitivity C-Reactive Protein, IBDQ = Inflammatory Bowel Disease Questionnaire, SES-CD = Simple Endoscopic Score-Crohn’s Disease, SHS = Simple Health Score.

**Figure 5.**
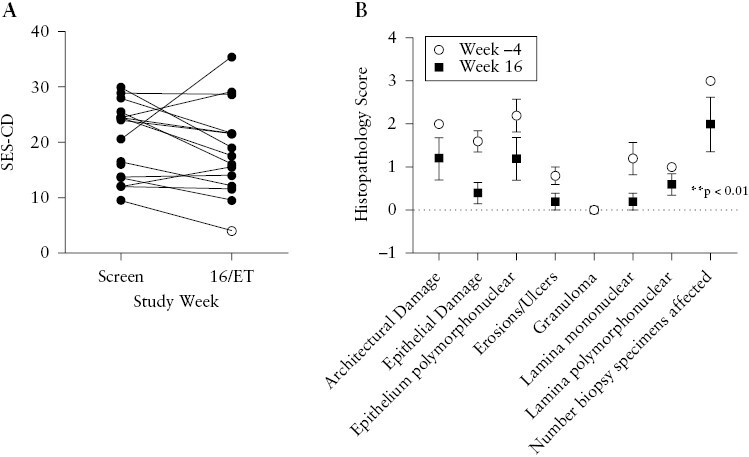
Bowel inflammation. [A] SES-CD for each patient at screening visit and Week 16/Early Termination visit. The hollow point denotes patient in SES-CD remission. [B] Histopathology subcomponent score in biopsies of the most affected region of the ileum [samples from six subjects; mean ± SEM]. Ileal histopathology change from baseline was analysed by restricted maximum likeliness [REML]. * *p* <0.01. SES-CD, Simple Endoscopic Score Crohn’s Disease; ET, early termination; SEM, standard error of the mean.

A decrease in SES-CD was observed from (median [interquartile range [IQR]) 24 [13.5–25.5] at Baseline to 17.5 [12–21.5] at Week 16, and numerically in 11/15 [73%] of patients with paired endoscopies [Figure 5A]. Two patients [13.3%] had ileal-colonic sections that were not scored at baseline yet scored at Week 16, artificially reducing improvement in SES-CD. One patient [7%] achieved endoscopic remission [all observed segments had an ulcer score of </= 1] at Week 16. Six patients [40%] and one patient [7%] had a decrease in SES-CD of >25% and >50%, respectively [Supplementary Figure 3A]. There were modest numerical improvements between Baseline and Week 16 in the mean SES-CD subscores in the sigmoid and left colon and in the terminal ileum [Supplementary Figure 3B]. In patients who achieved a clinical response at Week 16, there was a significant improvement in SES-CD subscores across the entire colon [*p *= 0.038] [Supplementary Figure 3C]. Ileal biopsies showed significant improvement from baseline at Week 16 across the eight histopathological subcategories [*p* <0.01] [Figure 5B]. However, histopathology of biopsies from the colon and the rectum did not show significant improvement from baseline [*p* = 0.14 and 0.93, respectively] [Supplementary Table 2].

**Figure 6.**
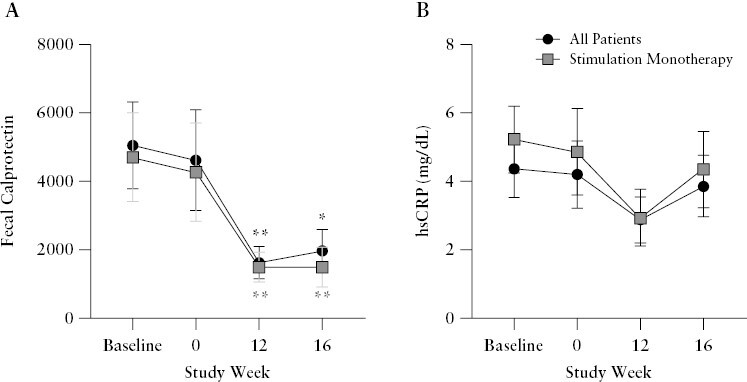
Disease-related biomarkers. [A] Faecal calprotectin [mean ± SEM] and [B] serum hsCRP [mean ± SEM] over time in the All Patients Efficacy population and in the 12-patient Stimulation Monotherapy subpopulation. Change from baseline was analysed by paired restricted maximum likeliness [REML] and adjusted with Bonferroni’s multiple comparisons test. *p <0.05, **p <0.01. hsCRP, high-sensitivity C-reactive protein; SEM, standard error of the mean.

A significant mean reduction from baseline in faecal calprotectin at Week 16 was observed in the All Patients group ([mean ± SEM] Baseline: 5054 ± 1266, Week 16: 1969 ± 625.5, *p* = 0.02) and in the Monotherapy group ([mean ± SEM] Baseline: 4705 ± 1295, Week 16: 1496 ± 579, *p* = 0.004) [Table 2, Figure 6A, Supplementary Figure 4A and B]. The mean level of faecal calprotectin over time is plotted in Figure 6A and shows stable levels from the pre-implantation biomarker baseline [the average of Week -4 and Week -2] to Week 0 when daily stimulation of the vagus nerve commenced. The mean faecal calprotectin level was significantly lower than baseline by 12 weeks of stimulation. Mean levels of CRP were numerically lower from Week 12 onwards compared with baseline [Figure 6B]. The reduction in faecal calprotectin was significant [*p* = 0.02] and the reduction in CRP trended lower [*p *= 0.20] in those patients who achieved a clinical response [Supplementary Figure 7].

The majority of patients reported improvement in their IBDQ [change in IBDQ >0] at Week 16, with 6 of 11 Stimulation Monotherapy patients exceeding the ‘minimal important difference’ of 16 [Table 2, Supplementary Figure 5A]. In contrast to the IBDQ, the SHS decreases as patients improve. By Week 16, a majority of patients reported improvements in their SHS compared with baseline [change in SHS <0] [Table 2, Supplementary Figure 5B]. In those patients who achieved a clinical response, the improvements in both IBDQ and SHS were significant [*p* <0.05] [Supplementary Figure 7].

**Figure 7.**
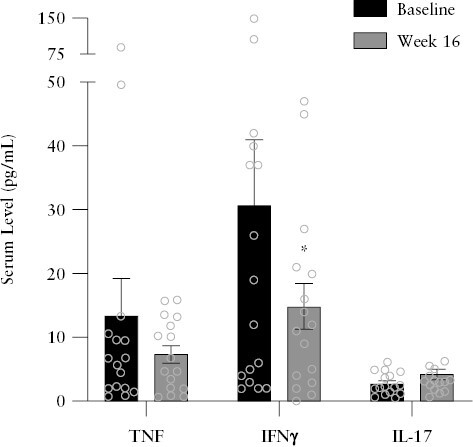
Serum cytokines: TNF, IFN-γ, and IL-17 [mean ± SEM]. Change from baseline was analysed by paired Wilcoxon test. *p <0.05. SEM, standard error of the mean; TNF, tumour necrosis factor; IL, interleukin.

Serum cytokine concentrations were also measured to assess the inflammatory biomarker response to vagus nerve stimulation. We observed a 46% and 52% decrease from baseline in mean levels of TNF and IFN-γ [Figure 7, Supplementary Figure 6]. Mean total Il-17 levels were 54% higher at Week 16 than at baseline. The full cytokine panel is presented in Supplementary Table 3. Correlations of the change from Baseline to Week 16 in clinical [CDAI], molecular [faecal calprotectin, hsCRP, TNF, IFN-γ, IL-17], endoscopic [SES-CD], and quality of life outcomes [IBDQ, SHS] were analysed to investigate the interactions between the varied endpoints in the context of this therapy [Supplementary Figure 8A and B]. Primarily, the change in CDAI was significantly correlated to changes in the quality-of-life assessments, and it positively correlated to the change in SHS and negatively correlated to the change in IBDQ [Spearman r >|0.5|, *p* <0.05] [Supplementary Figure 6A and B]. Additional interactions are described in Supplementary Figure 6. In those patients who achieved a clinical response, changes in serum levels of TNF, IL-17, and IFN-γ move together with a significant correlation between changes in IL-17 and IFN-γ levels [Spearman r = 0.8, *p* = 0.02] [Supplementary Figure 8 C and D].

### 3.3. Safety

All 17 patients reported at least one treatment-emergent adverse event during the study and most were mild or moderate [Table 3]. Eight patients experienced at least one SAE. All but one event was CD related and 50% of these SAEs [6/12] occurred prior to initiation of stimulation on Day 0. None were deemed to be treatment or device related, and one was related to device implantation/explantation [Supplementary Table 4]. Three patients discontinued the study prematurely, including one patient with a postoperative wound infection following device implantation [the implantation-related SAE]. One patient withdrew; their device was removed to enable diagnostic MRI. Another patient developed a relapse of Crohn’s disease with a prolonged, increased, inflammatory response. No significant adverse vital signs, physical examination or other observational findings were noted.

**Table 3.** Treatment-emergent adverse events occurring in > 10% of patients enrolled in the study

Adverse event	Safety Population, *n* = 17, *n* [%]
Crohn’s disease exacerbation	7 [41.2]
Abdominal pain	3 [17.6]
Anaemia	3 [17.6]
Pyrexia	3 [17.6]
Cachexia	2 [11.8]
Hypokalaemia	2 [11.8]
Pallor	2 [11.8]
Dysphonia	2 [11.8]
Oropharyngeal pain	2 [11.8]
Alopecia	2 [11.8]
Back pain	2 [11.8]
Joint swelling	2 [11.8]
Pain in jaw	2 [11.8]
Fatigue	2 [11.8]
Any serious adverse event	8 [47]
Any serious infection	2 [11.8]
Any cancer	0 [0]

## 4. Discussion

In this 16-week, open-label, clinical trial of neuroimmune modulation therapy in 17 patients with moderately to severely active CD, we observed a significant reduction in CDAI and faecal calprotectin levels. Clinical response [decrease in CDAI of at least 70 or 100 points], and clinical remission were achieved in a substantial percentage of patients. Together, these data indicate that vagus nerve stimulation improves CD clinical activity within 8 to 16 weeks. Moreover, some improvements in endoscopic severity were observed, even though this did not reach statistical significance. Significant endoscopic improvements were observed in the colons of those patients who achieved a clinical response. Significantly improved histopathological outcomes at Week 16 were also observed in the terminal ileum, but not in colonic segments, based on the Geboes score of paired biopsies with histopathological inflammation at baseline. There were no treatment-related serious adverse events. One implantation-related infection was reported.

The vagus nerve has immunoregulatory functions, and one important mechanism is the inflammatory reflex. Vagus nerve stimulation has been demonstrated to reduce inflammation in various colitis and intestinal inflammation models, including DSS-, oxazolone-, and TNBS-colitis, indomethacin enteropathy, and post-operative ileus.^18,30–35^ Electrical stimulation of the cervical vagus nerve reduces serum TNF and attenuates the severity of sepsis through a mechanism requiring cholinergic inhibition of pro-inflammatory immune cells.^47–49^ The vagus nerve innervation of the gut is still not completely mapped, and although vagus nerve innervation of the small bowel is well established, there are conflicting reports as to whether the colon is directly innervated.^50–52^ Cholinergic nerve endings have been localised adjacent to the myenteric plexus of the intestinal wall, but details of the interaction between intestinal immune cells and the vagus nerve are incompletely understood^53–55^ [Figure 1].

A number of studies have focused on nicotinic ACh receptor-mediated regulation of gut inflammation and vagus nerve stimulation in murine colitis models. These results indicate that the vagus nerve regulates colonic inflammation and that stimulating the vagus nerve attenuates gut-specific disease activity.^30,32,56,57^ A recent published study also reported reduction of small bowel inflammation by stimulating the vagus nerve of rats in a model of Crohn’s-like disease, through a spleen-independent mechanism.^34^ The immunoregulatory role of the vagus nerve in the gut has been studied in humans, and one epidemiological study looked at 15 637 vagotomised patients, finding a significant association between vagotomy and later development of CD with an incidence of 0.38 per 1000 person-years compared with 0.26 in non-vagotomised controls.^58^ Vagus nerve stimulation has been used as a therapy for refractory epilepsy for 25 years, and vagus nerve stimulation devices have been implanted in more than 125 000 patients, with high patient tolerability.^8,10^ The independent pilot vagus nerve stimulation study expanded to nine patients with moderately active CD naïve to biologic drugs, and report at 12 months has shown encouraging data on the reduction of symptoms and inflammatory biomarkers as well as endoscopic improvement, also reporting no serious adverse events.^37^

Observational studies in humans support the role of the vagus nerve and the parasympathetic tone in gut inflammation. One study reported lower vagal tone, as measured by heart rate variability, in CD patients compared with healthy controls, and another report showed that patients with high resting vagal tone had lower circulating TNF.^59,60^ Furthermore, in patients with ulcerative colitis, an association was observed between higher parasympathetic activity during a flare of the disease and lower systemic inflammation during a 3-year follow-up.^61^ An association has also been reported between a history of vagotomy and the development of CD.^58^

The first human study of an implanted vagus nerve stimulation device to report inhibition of cytokine biomarkers was performed in seven patients under full anaesthesia during implantation of a vagus nerve stimulator for the treatment of epilepsy. The data showed that endotoxin-induced TNF production in blood drawn from the patients was significantly reduced after a single intraoperative stimulation.^39^ Several small human trials of vagus nerve stimulation for inflammatory diseases, with promising efficacy outcomes, were reported, including: an open-label multicentre trial of 17 patients with active rheumatoid arthritis refractory to methotrexate and/or multiple biologic agents; a double-blinded multicentre trial of 14 patients with active rheumatoid arthritis refractory to multiple biologic and/or targeted synthetic agents; and an open-label, single-centre trial of nine patients with CD.^36–39^ The vagus nerve stimulation dosing in the rheumatoid arthritis trials was similar to what was used in the current study. The electrical stimulation significantly decreased TNF concentrations in circulating blood and resulted in significant improvement of clinical signs and symptoms according to the standard disease activity and patient disability indices [DAS28-CRP and HAQ-DI, respectively].^38,39^ In inflammatory bowel disease [IBD], Sinniger *et al.* reported a 12-month study in nine biologic-naive CD patients with moderately active disease at entry. The same device as used in the current study was implanted on the left vagus nerve and the same vagus nerve stimulation protocol used clinically to treat drug-refractory epilepsy was applied, i.e. 30 s of stimulation every 5 min. The electrical amplitude was titrated according to individual tolerability, and seven patients reached the 52-week visit [two early terminations]. The cytokine levels normalised toward those seen in healthy controls, especially IL-6, IL-12/23, transforming growth factor β1 [TGF-β1], and TNF, and the patients displayed a reduction in clinical activity and endoscopic scores.^37^ Even with dosing repeated 12 times every hour, vagus nerve stimulation was well tolerated and without serious complications.

In contrast to the high number of daily doses delivered to the vagus nerve in the treatment of epilepsy and in the prior CD study, we restricted the electrical stimulation in the current study to just one to four times daily in sessions lasting 1–5 min. The anti-inflammatory effect of the limited periods of electrical stimulation in our study is supported by previous reports on durable resolution of inflammation from a short electrical stimulation of the vagus nerve. Translational evidence for an anti-inflammatory effect lasting 24–48 h comes from studies with α7 nicotinic ACh receptor agonism of primary human macrophages exposed to endotoxin in culture, with vagus nerve stimulation in mouse endotoxaemia, and with human rheumatoid arthritis.^38,39,62,63^ Importantly, rats injected with indomethacin, which causes small bowel mucosal inflammation, were protected for up to 48 h after a 60-s electrical stimulation of the vagus nerve.^34^ Limiting the frequency, duration, and strength of electrical charges will mitigate potential off-target effects [such as hoarseness and discomfort] caused by contraction of laryngeal muscles during stimulation. Less frequent stimulation also reduces the energy use, which can allow for smaller batteries and devices, thereby facilitating implantation and clinical usability.^64,65^

In this study, stimulating the vagus nerve appeared to be a relatively safe intervention. No major safety signals were detected during the 16-week trial and all adverse events were mild to moderate. One patient developed postoperative infection that was resolved after explantation of the device.

The strengths of this study are the rigorous objective assessment of the endpoints despite lack of a control group with a combination of clinical activity and quality of life indices, endoscopic and histological evaluation, and biochemical and cytokine analyses. The evidence for an efficacy signal was generally consistent across these different types of instruments. The limitations of the study are the relatively small number of patients, the refractoriness of the populations, and the open-label design. The modest extent of endoscopic healing at Week 16 did not match the robust improvement in clinical outcome and decrease in faecal calprotectin. A later measurement, perhaps at 6 months, might have provided stronger evidence of robust endoscopic healing. The lack of improvement in colonic histology was inconsistent with the endoscopic response, and may be due to the biopsy-sampling strategy employed in combination with a relatively short and clearly defined segment of terminal ileum, compared with the dispersed biopsies from the total length of the colon.

Overall, this study has demonstrated that device implantation and electrical stimulation of the vagus nerve for several minutes per day was generally safe and well tolerated in biologic-refractory patients with moderately to severely active CD. Vagus nerve stimulation treatment resulted in a clinically meaningful reduction of clinical disease activity, with associated improvements in quality of life, colonic inflammation, and levels of faecal calprotectin and serum cytokines. It should be noted that with this small study population, these results should be interpreted with caution and a larger, double-blinded, controlled clinical study is warranted.

## Supplementary Material

jjad151_suppl_Supplementary_Material

## Data Availability

Data requests may be submitted to SetPoint Medical, Inc., at [https://Setpointmedical.com] and must include a description of the research proposal.
