# Supplementary material for: Neuroimmune Modulation Through Vagus Nerve Stimulation Reduces Inflammatory Activity in Crohn’s Disease Patients: A Prospective Open-label Study
Source: J Crohns Colitis. 2023 Sep 21;17(12):1897–909. doi: 10.1093/ecco-jcc/jjad151 (PMC10798868; doi:10.1093/ecco-jcc/jjad151)
Supplement: jjad151_suppl_Supplementary_Material [file jjad151_suppl_supplementary_material.pdf]

Supplementary Tables and Figures

Supplementary Figure 1. The per patient change in CDAI over time

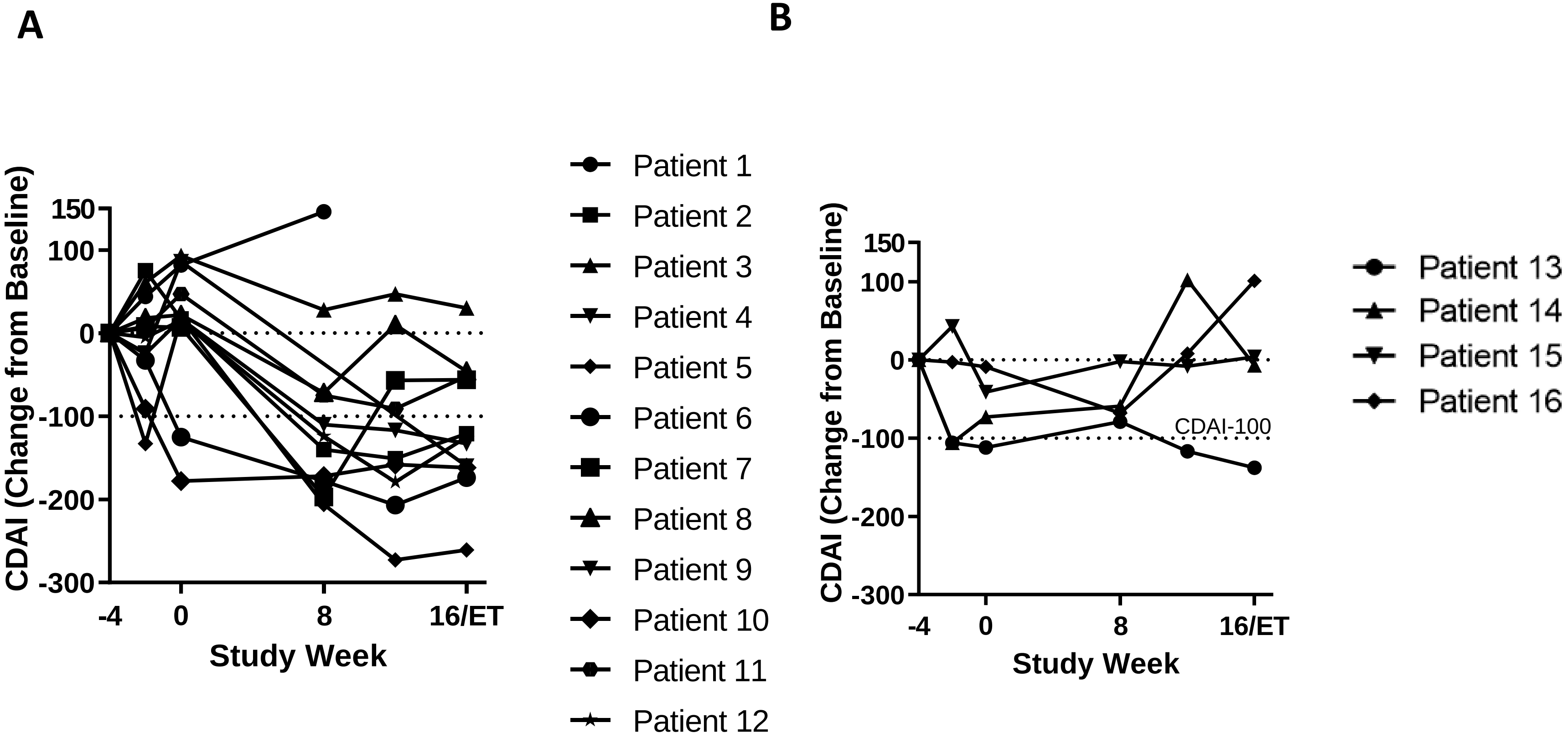

Supplementary Figure 1. Clinical efficacy. Per patient change from baseline in CDAI for **[A]** Patients treated with vagus nerve stimulation without biologics: “Stimulation Monotherapy group” and **[B]** Patients treated with vagus nerve stimulation and with biologics. Patient 13 was on steady dose of vedolizumab; Patient 14 was on a steady dose of infliximab; Patient 15 washed out infliximab during study, then added adalimumab; Patient 16 washed out infliximab during study. ET, Early termination.

Supplementary Figure 2. The per patient CDAI over time

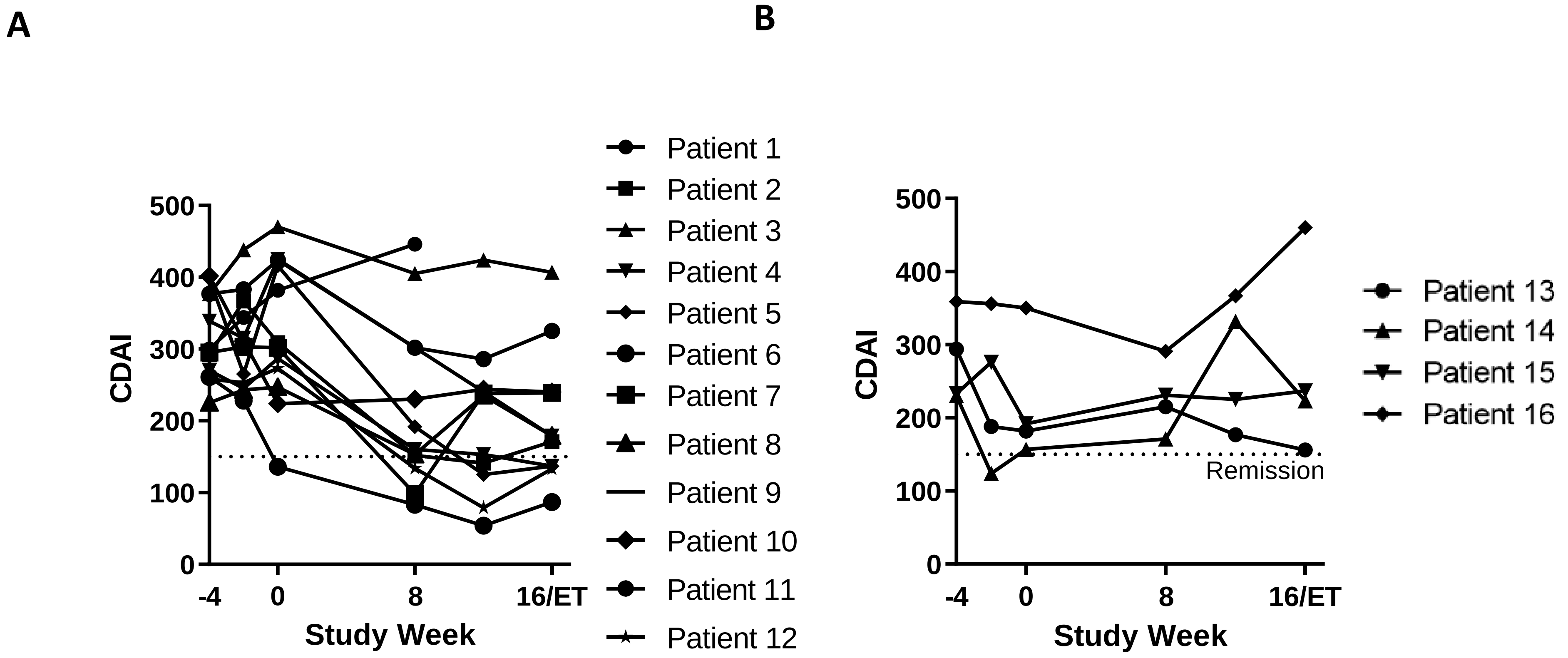

Supplementary Figure 2. Clinical efficacy. Per patient CDAI over time for **[A]** Patients treated with vagus nerve stimulation without biologics: “Stimulation Monotherapy group” and **[B]** Patients treated with vagus nerve stimulation and with biologics. Patient 13 was on steady dose of vedolizumab; Patient 14 was on a steady dose of infliximab; Patient 15 washed out infliximab during study, then added adalimumab; Patient 16 washed out infliximab during study. ET, Early termination.

Supplementary Figure 3. Endoscopic outcomes

A

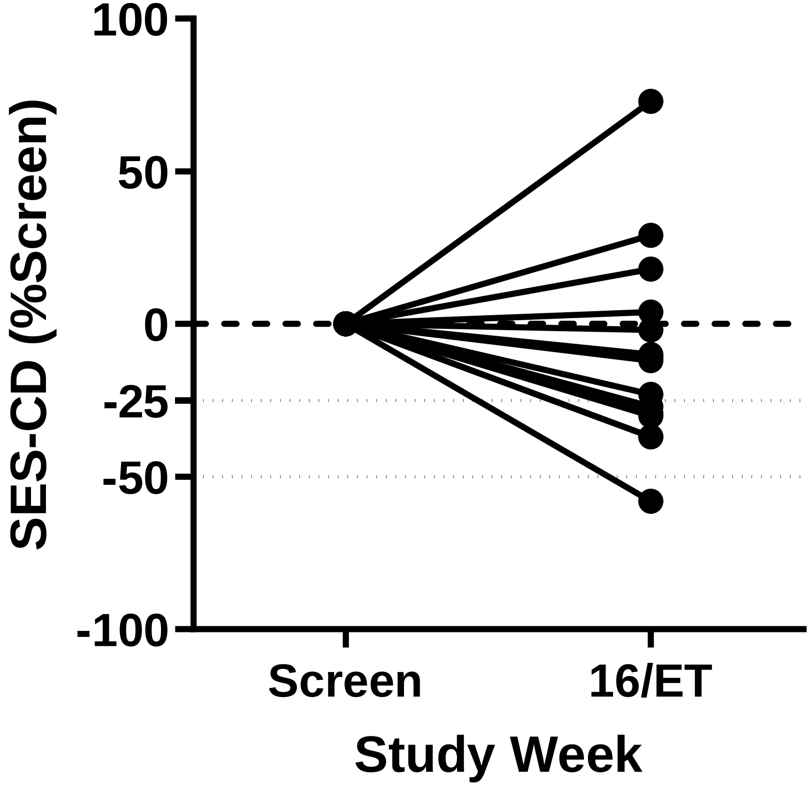

Supplementary Figure 3. Endoscopic outcomes. **[A]** % change from screening (baseline) SES-CD for each patient at screening visit and Week 16 / Early Termination visit. ET, Early termination.

Supplementary Figure 3. Endoscopic outcomes

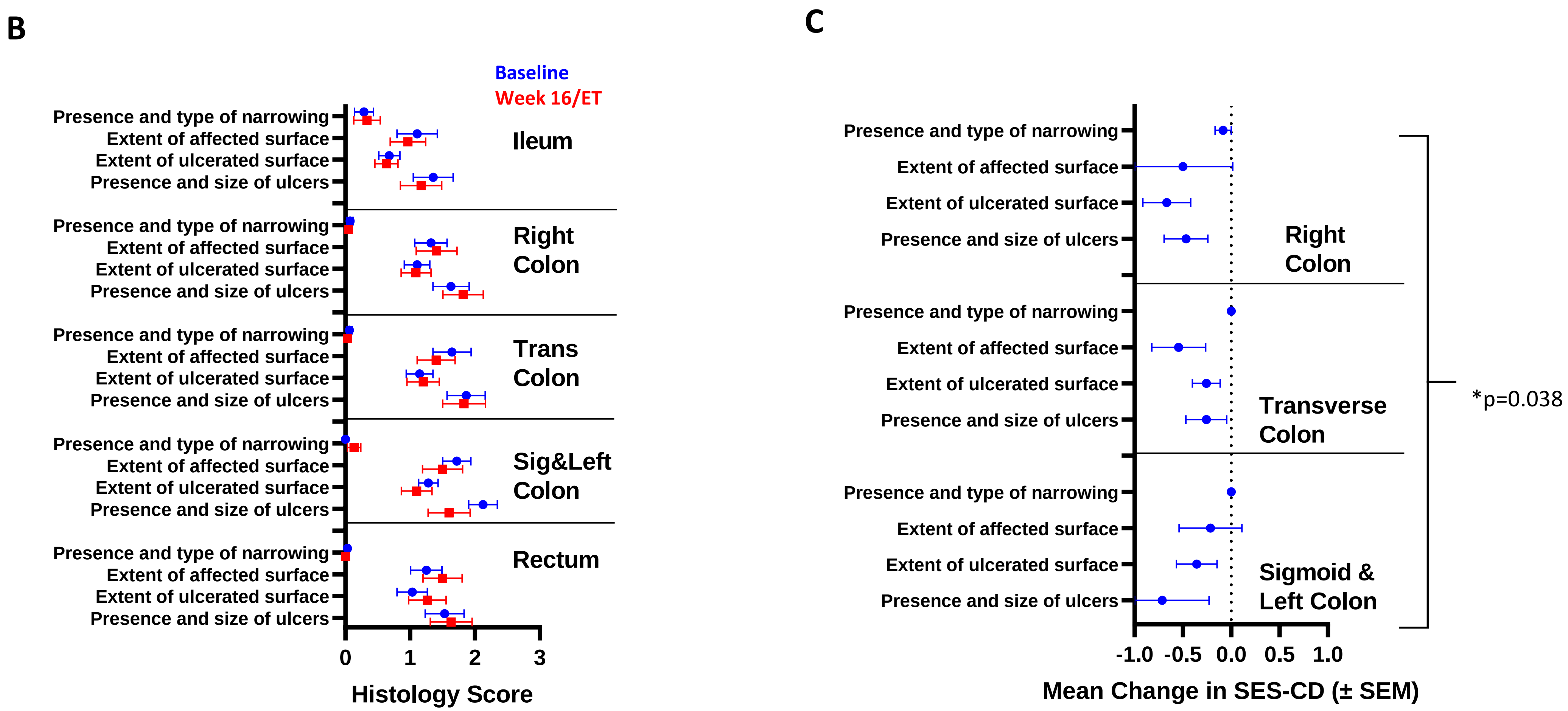

Supplementary Figure 3. Endoscopic outcomes. **[B]** SES-CD subscores of efficacy population (n=16) and **[C]** Change in colonic SES-CD subscores of patients that achieved CDAI-100 response (n=8). Data plotted as mean ± SEM. Endoscopic change from baseline was analyzed by REML. * $p<0.05$. SES-CD, Simple endoscopic score - Crohn's disease; ET, Early termination.

Supplementary Figure 4: Per Patient Changes in Faecal Calprotectin

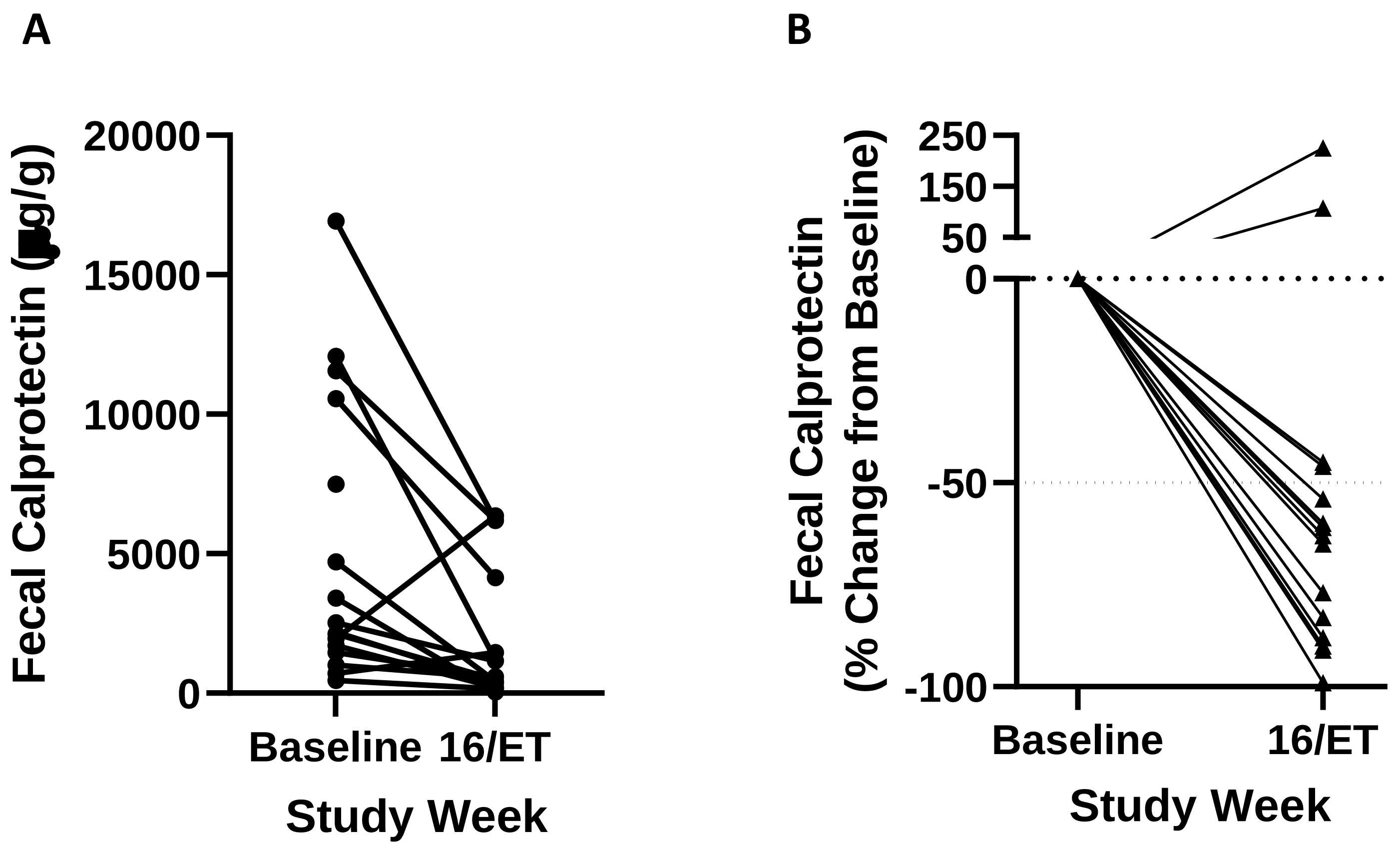

Supplementary Figure 4. Per Patient Changes in Faecal Calprotectin. **[A]** Change in faecal calprotectin **[B]** % Change from baseline in faecal calprotectin; ET, Early termination.

Supplementary Figure 5: Change in Disability Indices

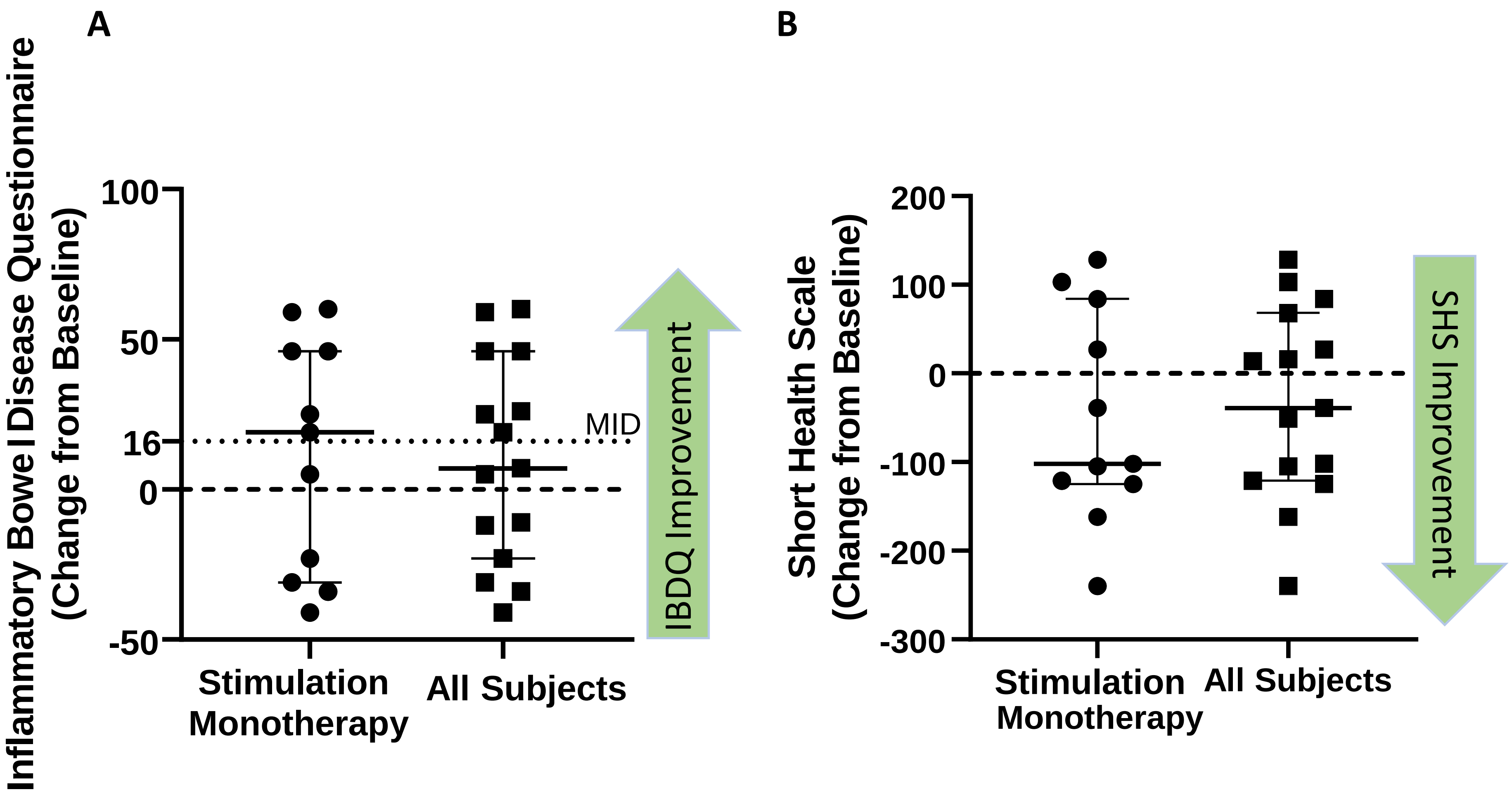

Supplementary Figure 5. Change in Disability Indices. **[A]** Change from baseline in the Inflammatory Bowel Disease Questionnaire and **[B]** Change from baseline in the Short Health Scale. Data plotted as median ± interquartile range.

Supplementary Figure 6: Per Patient Changes in Serum Cytokines

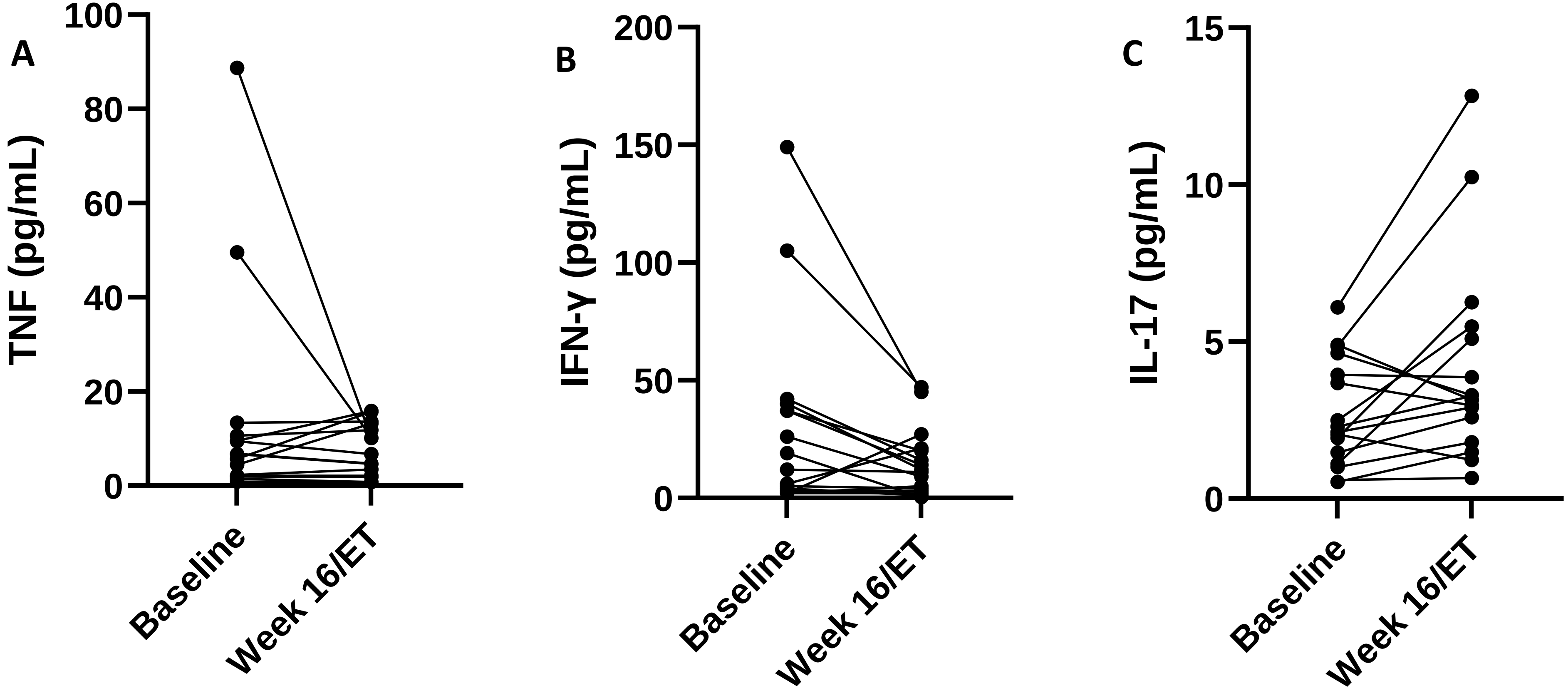

Supplementary Figure 6. Per Patient Changes in Serum Cytokines. **[A]** Change in TNF **[B]** Change in IFN-γ **[C]** Change in IL-17; ET, Early termination.

Supplementary Figure 7: Analysis Of Outcomes in CDAI-Responders

Clinical and Patient Reported Outcomes

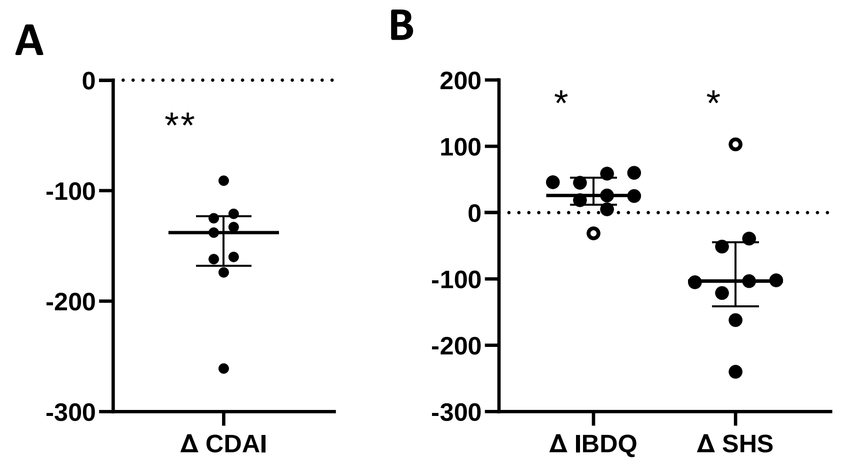

Objective Outcomes

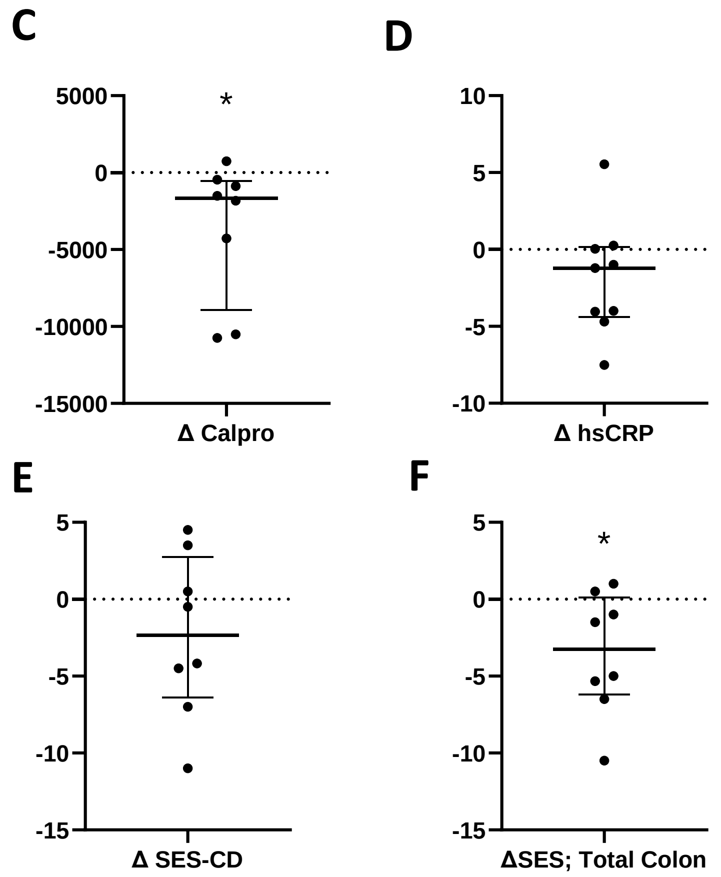

G

	Median	n	P value summary	P value (two tailed)
Δ CDAI	-138	9	**	0.004
Δ Calpro	-1664	8	*	0.023
Δ hsCRP	-1.205	9	ns	0.203
Δ SES-CD	-2.333	8	ns	0.313
ΔSES; Total Colon	-3.25	8	*	0.047
Δ IBDQ	26	9	*	0.039
Δ SHS	-103	9	*	0.031

Supplementary Figure 7. Analysis Of Outcomes in CDAI-Responders. Posthoc analyses of changes from baseline of select outcomes in patients with a CDAI-70 response at Week 12 or 16 (n=9) **[A]** Change in CDAI **[B]** Change in IBDQ and SHS. The hollow points represent the same subject and move against clinical response **[C]** Change in faecal calprotectin **[D]** Change in hsCRP **[E]** Change in SES-CD **[F]** Change in the total colon component of the SES-CD **[G]** Statistics table of analyzed outcomes. Wilcoxon column test was used to compare the median change from baseline with a hypothetic change of 0. *p<0.05, **p<0.01. Data plotted as median ± interquartile range.

Supplementary Figure 8A-B. Spearman correlation heatmap across clinical, molecular, endoscopic, and quality of life outcomes.

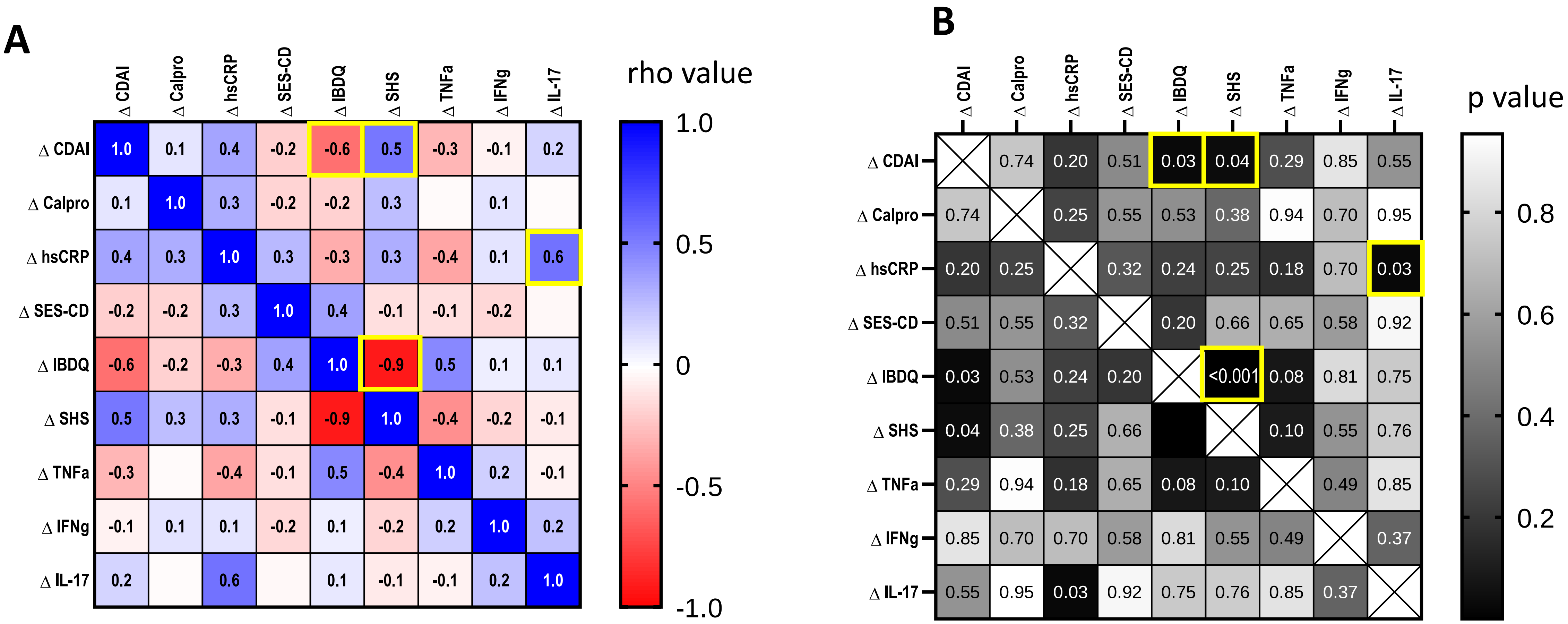

Supplementary Figure 8C-D. Spearman correlation heatmap of the change in serum cytokines levels in patients that achieved a CDAI-100 response.

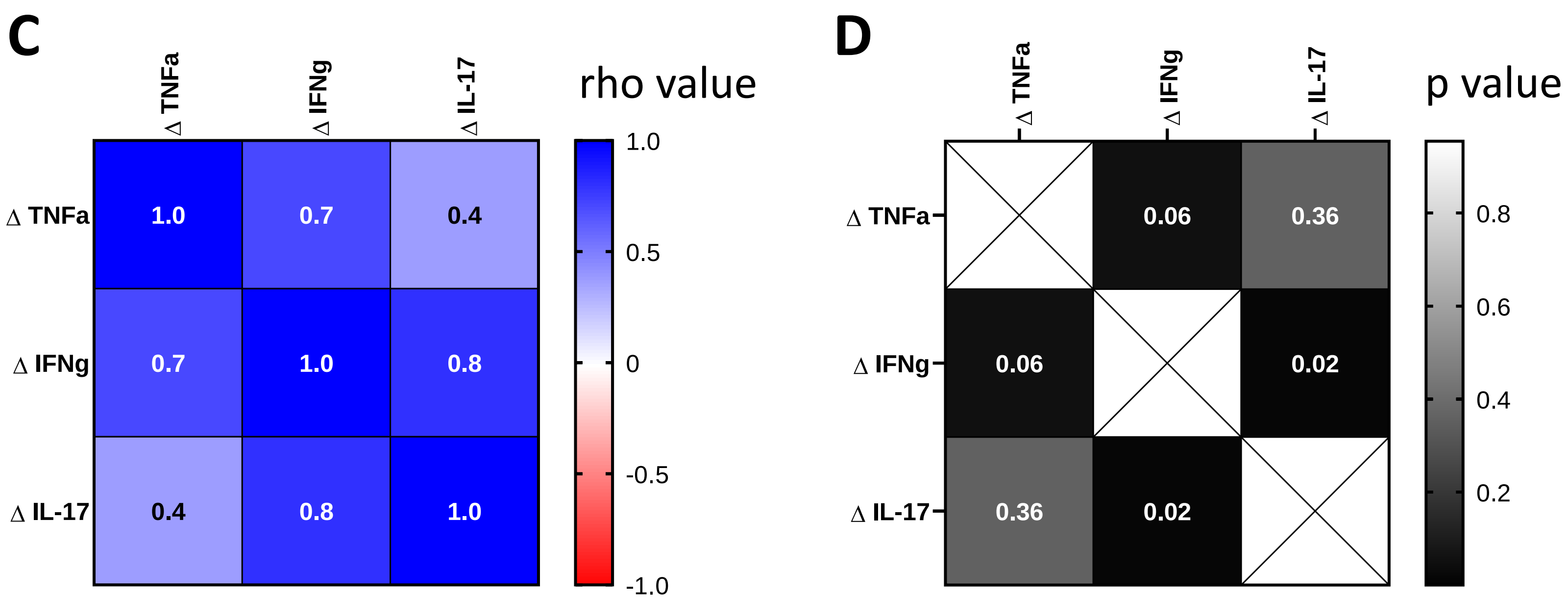

Supplementary Figure 8. Spearman correlation heatmaps [A-B] Spearman correlation heatmap across clinical, molecular, endoscopic, and quality of life outcomes**.** The change in CDAI was significantly correlated to changes in the quality-of-life assessments: positively correlated to the change in SHS and negatively correlated to the change in IBDQ (Spearman r > |0.5|, $p < 0.05$). As expected, the changes in SHS and IBDQ were strongly negatively correlated (Spearman r = -0.9, p = 0.000). There was a similar, but weaker correlative trend in changes in TNF levels and in SHS and IBDQ (Spearman r > |0.4-0.5|, $p < 0.1$). Changes in hsCRP and IL-17 were significantly correlated (Spearman r = 0.6, p = 0.03) as were the percent change from baseline in calprotectin and hsCRP (Spearman r = 0.8, p = 0.001), not shown. Yellow boxes highlight the cells where p <0.05. **(C-D)** Spearman correlation heatmap of the change in serum cytokines levels in patients that achieved a CDAI-100 response. The change in serum levels of TNF, IL-17, and IFN-γ move together in subjects that achieved a CDAI-100 response at Week 16. The correlation between IL-17 and IFN-γ was significant, while all other interactions trended to or approached significance.

Supplementary Table 1. Inclusion and Exclusion Criteria

Inclusion Criteria

Patients must meet all of the following inclusion criteria in order to be eligible for enrollment in this study:

- Male or female Patients aged 18-75 years, inclusive
- Written informed consent prior to any of the screening procedures
- Diagnosis of Crohn's disease for more than 4 months prior to Week -4 Visit, with small bowel and/or colonic involvement
- Current evidence of moderately-to-severely active disease defined by a Week -4 Visit Crohn's Disease Activity Index (CDAI) score of 220 to 450, inclusive
- Simple Endoscopic Score for Crohn’s Disease evaluation at baseline showing presence of a minimal ulcer score of 2 or 3 in at least 1 segment
- Levels of fecal calprotectin greater than or equal to 200 microgram/gram feces at Week -4 Visit
- History of inadequate response and/or intolerance or adverse events to one or more TNF-alpha inhibitors (e.g., infliximab, adalimumab, or certolizumab pegol), or vedolizumab.
- Female Patients of child-bearing potential are eligible if not pregnant, not planning to become pregnant during the course of the study, and committed to use of contraceptive methods with a failure rate of less than 1 percent per year

Exclusion Criteria

Patients who meet any of the following criteria are not to be enrolled in this study:

- Celiac disease
- Diagnosis of ulcerative or indeterminate colitis
- Enterocutaneous, abdominal or pelvic fistulae with abscesses, or fistulae likely to require surgery during the course of the study period
- Bowel surgery, other than appendectomy, within 12 weeks prior to Week -4 Visit and/or has planned surgery or deemed likely to need surgery for Crohn's disease during the study period

Exclusion Criteria continued

- Extensive colonic resection, subtotal or total colectomy
- Presence of ileostomies, colostomies or rectal pouches
- Fixed symptomatic stenoses of small bowel or colon
- History of more than 3 small bowel resections or diagnosis of short bowel syndrome
- Use of prohibited medications inside the specified washout period (prior to Week -4 Visit), and throughout the study. Prohibited medications include the following:
    - TNF antagonists and vedolizumab may continue throughout the study, but treatments should have been given at a stable dose for at least 6 months prior to the screening date and should be maintained at this level throughout the study
    - Use of natalizumab within 8 weeks
    - Use of glucocorticoids at doses greater than 10 mg prednisone orally QD, or an equivalent dose of other oral or parenteral glucocorticoids within 4 weeks
    - Use of cyclosporine, tacrolimus, sirolimus or mycophenolate mofetil within 4 weeks
    - Use of intravenous antibiotics for Crohn's disease within 4 weeks
    - Use of parenteral, tube or enteral feeding, or elemental diet within 2 weeks
    - Rectal Treatment: Use of 5-aminosalicylates or corticosteroid enemas or suppositories within 2 weeks
    - Azathioprine, 6-mercaptopurine and methotrexate can be continued throughout the trial. These medications must have been used for >12 weeks, at stable dose for at least 3 weeks prior to the Week -4 Visit.
- Leukocytopheresis or granulocytopheresis within 2 weeks prior to Week -4 Visit
- Positive immunoassay for *Clostridium difficile* at Week -4 Visit
- Known HIV infection
- Known active in infection with HBV or HCV
- Current evidence of, or has been treated for a malignancy within the past five years (other than localized basal cell or squamous cell skin cancer, cervical dysplasia, or any cancer which has been fully staged as *in situ* and has been fully resected)

Exclusion Criteria continued

- History of evidence of adenomatous colonic polyps that have not been removed.
- Use of any investigational product within 30 days prior to Week -4 Visit for small molecules, or 8 weeks prior for monoclonal antibodies
- Significant psychiatric disease or substance abuse
- History of unilateral or bilateral vagotomy
- History of recurrent vaso-vagal syncope episodes
- Known obstructive sleep apnea
- Known history of cardiac rhythm disturbances, atrio-ventricular block of greater than first degree, or cardiac conduction pathway abnormalities other than isolated right bundle branch block or isolated left anterior fascicle block. Evaluation by a cardiologist is required if the family history, patient history, or electrocardiogram suggests an abnormal cardiac conduction pathway.
- Significant pharyngeal dysfunction or swallowing difficulties
- Pre-existing clinically significant vocal cord damage or hoarseness
- Previously implanted electrically active medical devices (e.g., cardiac pacemakers, automatic implantable cardioverter-defibrillators)
- Asthma or chronic obstructive pulmonary disease not controlled by medications, or any other disease causing clinically significant dyspnea at time of screening
- A greater than or equal to 40 pack-year smoking history
- Active peptic ulcer disease
- Patients with a limited life expectancy due to terminal illness

Supplementary Table 2. Change in Geboes Subcategories

Histopathological Subcategories	Ileum			Global Colon			Rectum		
	mean	std	95% CI	mean	std	95% CI	mean	std	95% CI
**Architectural Damage**	-1.0	1.2	-2.8 - 0.8	0.1	0.3	-0.1 - 0.2	-0.3	0.7	-0.8 - 0.3
**Epithelial Damage**	-1.0	0.8	-2.3 - 0.3	0.1	1.0	-0.5 - 0.7	-0.3	1.0	-1.1 - 0.6
**Epithelium polymorphonuclear**	-0.8	1.7	-3.5 - 2	0.3	1.1	-0.4 - 1	0.1	0.8	-0.6 - 0.8
**Erosions/Ulcers**	-0.8	0.5	-1.5 - 0	-0.1	0.6	-0.5 - 0.2	-0.1	0.6	-0.7 - 0.4
**Granuloma**	0.0	0.0	- - -	0.1	0.4	-0.1 - 0.4	0.0	0.5	-0.4 - 0.4
**Lamina mononuclear**	-1.0	1.2	-2.8 - 0.8	0.0	1.0	-0.6 - 0.6	-0.5	0.9	-1.3 - 0.3
**Lamina polymorphonuclear**	-0.5	0.6	-1.4 - 0.4	0.0	0.6	-0.3 - 0.3	0.1	0.6	-0.4 - 0.7
**Number biopsy specimens affected**	-1.3	1.5	-3.6 - 1.1	0.2	0.4	0 - 0.4	-0.4	0.7	-1 - 0.2
**REML p-value (Study Visit)**		<0.01			0.136			0.934	

Supplementary Table 3. Serum cytokine

Analyte	GM-CSF	IL-12 p40	IL-15 human	IL-16	IL-17	IL-1 alpha	IL-5	IL-7	TNF beta	VEGF
Baseline mean (pg/mL)	0.5	71.7	4.8	151.6	2.7	1.7	0.4	25.4	0.5	895.3
Baseline SD (pg/mL)	1.0	68.8	5.3	171.9	1.7	2.0	0.7	13.7	0.7	1548.9
Week 16 mean (pg/mL)	0.5	124.0	4.7	154.1	4.2	2.5	0.3	32.3	0.1	1616.5
Week 16 SD (pg/mL)	0.8	112.0	5.8	142.7	3.3	3.4	0.8	22.3	0.2	4201.1
Analyte	IFN-γ	IL-10	IL-12 p70	IL-13	IL-1β	IL-2	IL-4	IL-6	IL-8	TNF
Baseline mean (pg/mL)	30.7	2.4	2.3	3.6	0.1	53.6	0.0	5.3	19.3	13.4
Baseline SD (pg/mL)	41.3	4.5	1.8	5.0	0.1	211.2	0.0	5.8	17.0	23.2
Week 16 mean (pg/mL)	14.9	1.9	2.1	4.7	0.1	43.8	0.0	7.0	18.7	7.3
Week 16 SD (pg/mL)	14.6	1.5	1.9	6.4	0.1	171.7	0.0	7.2	11.7	5.6
Analyte	Eotaxin	Eotaxin-3	IL-8	IP-10	MCP-1	MCP-4	MDC	MIP-1α	MIP-1β	TARC
Baseline mean (pg/mL)	298.1	15.0	49.5	357.4	222.0	57.7	810.6	21.7	123.7	385.3
Baseline SD (pg/mL)	200.0	11.2	60.1	543.9	136.7	60.2	528.5	11.6	93.2	355.9
Week 16 mean (pg/mL)	266.8	18.7	55.7	262.9	233.9	62.7	1036.4	23.3	188.4	440.6
Week 16 SD (pg/mL)	184.7	11.5	47.5	203.2	162.4	71.7	749.2	11.3	211.4	573.3

Supplementary Table 4. Severe Adverse Events

Subject ID	Event Preferred Term	Outcome	Severity	Causality	Action taken on Device
A	Crohn's disease	RWS	Moderate	Not Related	None
	Gastroenteritis viral	RWS	Severe	Not Related	None
B	Ileus	RWS	Severe	Not Related	Other: NA
	Crohn's disease	RWS	Severe	Not related	Other: NA
C	Dehydration	RWS	Severe	Not Related	None
	Prerenal failure	RWS	Severe	Not Related	None
D	Crohn's disease	RWS	Severe	Not Related	None
E	Inflammation	RWS	Severe	Not Related	Discontinued
	Cachexia	RWS	Severe	Not Related	Discontinued
F	Crohn's disease	RWS	Moderate	Not Related	Implantation of device has been delayed
G (Safety population)	Post-operative wound infection	RWS	Moderate	Implantation or explantation procedure-related	Removed
H	Crohn's disease	RWS: disease ongoing at Week 16	Moderate	Not Related	None

RWS: Recovered with sequalae
